# Knowledge, Awareness, Attitude and Preventive Behaviour on the Transmission of the Pandemic Novel Coronavirus Among Malaysians

**DOI:** 10.21315/mjms2021.28.2.10

**Published:** 2021-04-21

**Authors:** Norsa’adah Bachok, Anis Kausar Ghazali, Rohayu Hami

**Affiliations:** 1 Unit of Biostatistics and Research Methodology, School of Medical Sciences, Universiti Sains Malaysia, Kubang Kerian, Kelantan, Malaysia; 2 Advanced Medical and Dental Institute, Universiti Sains Malaysia, Kepala Batas, Pulau Pinang, Malaysia

**Keywords:** COVID-19, coronavirus, infection control, pandemic

## Abstract

**Background:**

Restricted movement and preventive actions have been introduced to break the chain of transmission of a new coronavirus. This study was conducted to determine the level of knowledge, awareness, attitude and preventive behaviour regarding the transmission of the COVID-19.

**Methods:**

A cross-sectional study was conducted among Malaysians aged 20 years old and over, who had accessed the internet and used the WhatsApp application. The sampling method was a convenient snowball from 14 Malaysian states. This study was conducted online using questionnaires during the Movement Control Order due to the pandemic.

**Results:**

Approximately 40.0%, 46.1% and 47.4% of 1,290 participants had a score above the median (good score) for preventive behaviour, attitude and knowledge, respectively. Age was significantly associated with poor knowledge (adjusted odds ratio [AOR] 0.98; 95% confidence interval [CI]: 0.97, 0.99; *P* = 0.026). Males (AOR 1.35; 95% CI: 1.05, 1.74; *P* = 0.021), Malays (AOR 1.41; 95% CI: 1.01, 1.98; *P* = 0.043) and Chinese (AOR 2.19; 95% CI: 1.17, 4.13; *P* = 0.015) were the associated factors for poor attitude. Chinese ethnicity was significantly associated with poor preventive behaviour (AOR 2.80; 95% CI: 1.39, 5.61; *P* = 0.004).

**Conclusion:**

The level of knowledge, attitude and practices were high except for a few questions. The young, males and Malay and Chinese individuals need health education.

## Introduction

A novel coronavirus was first reported in December 2019 in Wuhan, China (1). The highly transmissible virus is called severe acute respiratory syndrome coronavirus 2 (SARS-CoV-2) (1, 2), which causes coronavirus diseases, and was later known as COVID-19; this virus can cause a number of illnesses ranging from mild flu-like symptoms to life-threatening pneumonia (2).

The virus spread to Malaysia when a group of seven Chinese arrived in Malaysia via Singapore in January 2020. The situation worsened when a series of clusters occurred, with the major cluster being the Seri Petaling *Tabligh* cluster. The cluster is linked to a religious gathering held on 27 February to 1 March 2020, with more than 10,000 participants from Southeast Asia. This was the largest cluster to date, with a total of 1,701 positive cases that accounted for 48% of all positive cases in Malaysia (3).

The virus has spread worldwide, becoming a pandemic, as declared by the World Health Organization on 11 March 2020 (4). To date, there have been more than 109 million confirmed cases worldwide with 2.41 million deaths. The number of confirmed cases worldwide has reached immeasurable levels, forcing the affected countries to be in a lockdown state, including Malaysia. It is a necessary intervention to stop or slow down the transmission of COVID-19 while minimising the economic, public and social impacts. On 16 February 2021, there were 266,445 confirmed cases in Malaysia, with 975 deaths (0.37%) (5).

Coronavirus is a new virus, the natural history of which is also new to most people. It is important to establish the level of knowledge of attitude towards and compliance with COVID-19 preventive actions. Raising public awareness of the transmission of COVID-19 and its symptoms will result in an increase in health-seeking behaviours as well as self-quarantine and the prevention of contacts. This study will help policy makers to identify the extent of Malaysians’ awareness on this new disease, and how well we are complying and accepting new regulations to prevent this pandemic. Therefore, this study was conducted to determine the level of knowledge, awareness, attitudes and preventive behaviours regarding the transmission of the new pandemic COVID-19 among Malaysians.

## Methods

The research design is a cross-sectional study. The study was conducted from April to May 2020. The source of population was those who have access to the internet and use the WhatsApp application. We included Malaysians aged 20 years old and over living in Malaysia and excluded those who were infected with COVID-19.

The sampling method was a convenient snowball sampling. Initially, we posted the survey invitation with the link to the Google Form via the WhatsApp application. The starting point was researchers’ contacts, such as relatives and friends. In the post, we asked each of the individuals who received the link to pass it on to all of their adult friends and relatives. Then, the link was spread to others’ contacts. We sampled the participants according to their state of residence. We included all Malaysians from 14 Malaysian states. Selangor state was the most populous state (5.46 million), followed by Johor (3.35 million) and Sabah (3.21 million) (6). Thus, participants from Selangor were sampled more than less populous states such as Perlis. This study was conducted online during the Movement Control Order due to the pandemic.

A new set of questionnaires was designed to record all of the information, based on the literature (7) and previous research of the H1N1 influenza epidemic in 2010, which had been validated (8). The newly developed questionnaires were closed-ended with predetermined responses that consist of:

- Sociodemographic characteristics such as age, sex, race etc.
- Seventeen questions on awareness and knowledge. The responses were ‘yes’ or ‘no’, and a score of 1 was given for the yes and 0 for no. The maximum score was 17 and we converted the scores into percentages.
- Eight questions on attitude regarding their susceptibility and intention to comply. The responses have 4 point Likert rating: ‘strongly agree’, ‘agree’, ‘disagree’ and ‘strongly disagree’. We gave score of 4 for strongly agree, 3 for agree, 2 for disagree and 1 for strongly disagree.
- Ten questions on level of compliance with prevention behaviour to stop the transmission of COVID-19. The responses have three ratings: ‘all the time’, ‘sometimes’ and ‘not at all’. We gave a score of 2 for ‘all the time’, 1 for sometimes and 0 for ‘not at all’.

These questionnaires were validated before use. The newly developed questionnaires were transformed into a Google Form. In the Google Form, voluntary participants had to sign in, which was limited themselves to only one attempt. Once respondents submitted the responses, they could not be edited or resubmitted. The Google Form was inaccessible after we received the required number of samples.

Household income was categorised according to the median economic group drawn by the Malaysian Department of Statistics (9). The poorest 40% (B40) of households in Malaysia had a median income of RM3,000, the middle 40% (M40) had a median income of RM6,275 and the richest 20% (T20) had a median income of RM13,148 (9).

### Statistical Analysis

After reaching a sufficient number of respondents, data from the Microsoft Excel application were retrieved and transferred to the SPSS software version 25. Descriptive statistics were drawn to summarise all of the data. Numerical data were presented as mean (SD) or median (inter quartile range [IQR]) according to their distribution of normality and categorical data were presented as frequencies (percentages).

The dependent variables were knowledge, attitude and preventive behaviour towards COVID-19 infection. The scores of responses were totalled and then categorised by the median score into two categories: good and poor level. The poor level was coded as ‘1’ and good level as ‘0’ in the data entry for SPSS. We first performed simple logistic regression, followed by multiple logistic regression for multivariable analysis to examine the association between sociodemographic characteristics and knowledge, attitude and behaviour. All variables that have *P*-values < 0.25 in the simple logistic regression were included in the building of the preliminary model. The forward likelihood ratio (LR) and backward LR methods were used for model selection. We had chosen the simplest and most parsimonious fit model. The assumptions were checked for linearity of the continuous independent variable, multi-collinearity, interaction and fitness of the final model. Results of the model were presented with adjusted odds ratio (AOR), a 95% CI and its corresponding *P*-value.

## Results

We included 1,290 participants in the final analysis. Table 1 shows that the mean age of participants was 42.2 years (SD 10.2) with 74.1% females. The majority were of Malay ethnicity, married with a degree education, working in the government sector and in the high-income group of T20. Only 7.1% had relatives or acquaintances who had tested positive for COVID-19.

Table 2 presents the responses to the questions on knowledge and awareness of COVID-19. Regarding the portal entry of coronavirus, 97.2% of the participants answered nose, 94.7% mouth and 77.0% eyes. Regarding the mode of transmission of the virus, more than 90% answered ‘yes’ for all of the statements except for the eating wild animals. Only 58.7% agreed that the coronavirus is transmissible through the consumption of wild animals. Most participants agreed that asymptomatic patients can transmit the virus, isolation can prevent transmission and quarantine should be implemented for 14 days for exposed individuals. Regarding the symptoms of COVID-19, most participants agreed that the symptoms were fever, cough and difficulty breathing. Only 57.3% and 60.1% agreed on the symptoms of lethargy and runny nose, respectively.

Table 3 shows the responses for attitude statements. Statements 1 and 2 were about the vulnerability of people to infection, more than half of who disagreed. The other statements asked about their attitudes towards preventive measures; most of them had positive attitudes.

Compliance with most preventive behaviours, such as staying at home and avoiding crowded places, were respected all of the time. Avoid touching the face and covering the mouth when sneezing or coughing with a tissue or handkerchief were only sometimes complied with. These were shown in Table 4. Table 5 presents a summary of the descriptive results of the questions regarding knowledge, attitudes and practices. When we categorised the scores using the median as cut off point, 40.0%, 46.1% and 47.4% of participants had a score above the median (good score), for preventive behaviour, attitude and knowledge, respectively.

Next, we performed several simple logistic regressions to evaluate the association between sociodemographic characteristics and knowledge, attitude and preventive behaviour. Table 6 shows that there were significant associations between age and M40 income and poor knowledge. There were significant associations between age, sex, ethnicity, education level and M40 income and poor attitude, as shown in Table 7. There were significant associations between ethnicity, marital status and poor preventive behaviour and practices, as shown in Table 8.

The results by multiple logistic regression are displayed in Table 9. There was no interaction between variables. The selected models showed an overall fit, with the *P*-values of the Hosmer-Lemeshow test being non-significant. Age was significantly associated with knowledge when the occupation was adjusted (AOR 0.98; 95% CI: 0.97, 0.99; *P* = 0.026). As the age increased by one year, there was a significant 2% reduction in the risk of poor knowledge. Sex and ethnicity were significantly associated with poor attitude. Males were 1.3-times more likely to have poor attitudes compared to females when age and ethnicity were adjusted for (AOR 1.35; 95% CI: 1.05, 1.74; *P* = 0.021). Malays had 1.4-times (AOR 1.41; 95% CI: 1.01, 1.98; *P* = 0.043) and Chinese had 2.2-times (AOR 2.19; 95% CI: 1.17, 4.13; *P* = 0.015) the risk of poor attitude compared to other ethnicities when sex and age were adjusted for. Ethnicity was also significantly associated with behaviour with the Chinese people having a risk that was 2.8-times higher than that of other ethnicities with regard to poor behaviour when occupation was adjusted for (AOR 2.80; 95% CI: 1.39, 5.61; *P* = 0.004).

## Discussion

Most of our participants had higher education, were employed and were from the top 20% of household incomes. These characteristics corresponded to people who had access to the internet and smartphones. Smartphone users in Malaysia increased from 75.9% in 2017 to 78.0% in 2018 (10). Smartphone users who access the internet through their smartphones are growing at an average annual growth rate of 5.45%. In 2018, 94.6% of smartphone users were using their phones to go online. This provides an excellent opportunity to educate people through electronic media and social networks such as WhatsApp, Twitter and Facebook.

Most of our participants were aware of and knowledgeable about COVID-19. This indicated that the participants actively read the news and policies from the government. Among the knowledge statements, only 77.0% agreed on the eyes as a portal of entry, while only 57.3% and 60.1% agreed on lethargy and runny nose as symptoms of COVID-19, respectively. These are the aspects that need emphasis in COVID-19 education for the public.

Most participants were aware of the mode of transmission of the virus, as more than 90% answered yes for all of the statements. The main mode of transmission of COVID-19 is direct person to person contact and through respiratory droplets when someone speaks, coughs or sneezes (11, 12). It can also be transmitted when people are in close contact of one metre with each other. The droplets can then be inhaled, or can land on surfaces, which others may come into contact with, and who can then be infected by touching their nose, mouth or eyes. The virus can survive on inanimate surfaces like metal, glass or plastics from several hours to a few days, thus providing a source of continuous transmission if they were not cleaned regularly (13).

Only 58.7% agreed that the COVID-19 virus is transmissible through the consumption of wild animals. This is a controversial issue. There is a zoonotic linkage with SARS-CoV-2 and animals such as bats, civets and pangolins (1, 2, 14). It was concluded that there was no scientific evidence to support the animal-human connection, although there was a strong suspicion.

There are several symptoms and complications caused by the virus, including fever, headache, cough, tiredness, dyspnoea, myalgia and anosmia, with some individuals developing acute respiratory distress syndrome about a week after the onset of the disease, which can lead to death (1). There is evidence suggesting that transmission can be caused by asymptomatic carriers (15). Most of our respondents were aware of this fact, therefore they were cautious of viral exposure when meeting people unknown to them. Nasopharyngeal swabs were obtained from 210 pregnant women admitted for delivery in New York who were asymptomatic for COVID-19, with 13.7% found to be positive for SARS-CoV-2 (16). A screening in Iceland reported 13.3% positive results for SARS-CoV-2 among persons at high risk for infection such as symptomatic individuals, and those who had recently travelled to high-risk countries or had been in contact with infected persons. In contrast, 0.8% positive results were reported among the general population from open-invitation screening and 0.6% in the random-population screening (17).

A positive attitude can improve the outcomes of COVID-19, which includes feeling vulnerable and having the intention and motivation to comply with the rules and regulation to prevent the transmission of COVID-19. The Health Belief Model states that individuals who feel vulnerable to a particular health problem will adopt behaviours to reduce their risk of developing the health problem (18). More than half of our respondents disagreed with the attitude statements concerning the vulnerability of them to infection. On the other hand, most of our respondents agreed with statements regarding other intentions of complying with preventive behaviours.

The World Health Organization recommends important practices to reduce the risk of COVID-19 infection by washing hands frequently with soap and water, or by using an alcohol-based hand sanitiser, along with respiratory hygiene, maintaining the social distance of one metre and avoiding touching the eyes, nose and mouth (4, 7). The Ministry of Health Malaysia also recommends the use of face masks, which can prevent or reduce transmission if worn properly. The compliance of preventive behaviours, such as staying at home and avoiding crowded places was respected all the time by our respondents. Avoiding touching the face and covering the mouth with a tissue or handkerchief when sneezing or coughing were only sometimes complied with. Wearing face masks when having the flu was not practiced at all for some respondents. Wearing a face mask when having a flu whose status is unknown is a strategy to reduce public exposure to a possible COVID-19.

Older participants were significantly associated with poor knowledge. This is probably consistent with the fact that older people were less likely to engage with news posted in social media compared to younger people. In terms of attitude, males were more likely to have a poor attitude. Surprisingly, Malay and Chinese had a poor attitude compared to other ethnics in Malaysia. Other ethnics referred to ethnics in Sabah and Sarawak. The Chinese were at higher risk than other ethnicities for poor behaviour. There was no similar study found to compare with our results. However, a study on hepatitis reported that females and those with a doctorate education generally have better knowledge than the comparison group. Meanwhile, older age and married status were significantly associated with a better attitude and older age, being married and being females were significantly associated with good practices (19). In contrast, it was reported that older people, and those with a higher education and low self-perceived risk were more likely to have a positive attitude towards cancer information (20).

This study had large and representative samples of participants from all states in Malaysia. This study used an online method that is free from interviewer bias. A multivariable analysis was used to control the study confounders. However, there were several limitations of this study. Although our respondents were representative of all Malaysian states, our results showed that most of our respondents were in high education and income groups. For these reasons, our results showed a high level of knowledge, attitudes and practices. We used previously validated questionnaires of the H1N1 epidemic with some modifications. Since we wanted to conduct this study during the peak of the pandemic, we did not revalidate the questionnaires.

## Conclusion

The level of knowledge and awareness, attitude and practices was high except for a few questions. When we categorised them by median score, 40.0%, 46.1% and 47.4% of participants had poor scores for knowledge and awareness, attitude and practices, respectively. Young individuals, males, and Malay and Chinese ethnics need health education to increase their knowledge, attitudes and behaviours toward the prevention of COVID-19 in Malaysia.

Each individual in Malaysia plays an important role in breaking the transmission of COVID-19. We strongly support awareness and health promotion strategies as an integral part of a COVID-19 prevention and control programme.

## Figures and Tables

**Table 1:** Sociodemographic characteristics of participants (*n* = 1,290)

Variables	Frequency (%)	Mean (SD)
Age (years)		42.2 (10.2)
Sex		
Male	334 (25.9)	
Female	956 (74.1)	
Ethnicity		
Malay	1,053 (81.6)	
Chinese	56 (4.3)	
Indian	20 (1.6)	
Others	161 (12.5)	
Marital status		
Single	214 (16.6)	
Married	1,013 (78.5)	
Divorced/widow	63 (4.9)	
Education Level		
Primary school and less	18 (1.4)	
High school	146 (11.3)	
Diploma	227 (17.6)	
Bachelor	706 (54.7)	
Master/PhD	193 (15.0)	
Occupation		
Government sector	823 (63.8)	
Private sector	172 (13.3)	
Self-employed/odd job/others	99 (7.7)	
Housewife	69 (5.3)	
Student	61 (4.7)	
Retired/un-employed	66 (5.1)	
Family income (RM)		6,000 (6,200)*
T20	626 (48.5)	
M40	385 (29.8)	
B40	279 (21.6)	
Had relatives/friends with positive COVID-19	92 (7.1)	

Note:

* median (IQR)

**Table 2:** Knowledge and awareness about COVID-19 of participants (*n* = 1,290)

Variables	Frequency of ‘Yes’ (%)
Portal of entry of coronavirus:	
Eyes	993 (77.0)
Nose	1,254 (97.2)
Mouth	1,222 (94.7)
Mode of transmission of coronavirus:	
Unconsciously touching the eye, nose or mouth with a contaminated hand	1,263 (97.9)
Exposure to a respiration aerosol from a COVID-19 patient when talking, sneezing or coughing	1,254 (97.2)
Staying close, less than a metre from a COVID-19 patient	1,166 (90.4)
Touching any surfaces or doorknobs that are contaminated by a COVID-19 patient	1,194 (92.6)
Shaking hands with a COVID-19 patient	1,208 (93.6)
Eating wild animals such as bats	757 (58.7)
COVID-19 patients can spread disease even though they are healthy and show no symptoms	1,246 (96.6)
Symptoms of COVID-19:	
Fever	1,222 (94.7)
Coughs	1,242 (96.3)
Runny nose	775 (60.1)
Breathing difficulties	1,271 (98.5)
Lethargy	739 (57.3)
Isolation (quarantine) of COVID-19 patient can prevent the transmission of pandemic	1,284 (99.5)
A person exposed to a COVID-19 patient should be quarantined for 14 days	1,288 (99.8)

**Table 3:** Attitude on susceptibility and intention to comply to prevent the transmission of COVID-19 among Malaysians (*n* = 1,290)

Variables	Frequency (%)			

	Strongly agree	Agree	Disagree	Strongly disagree
I am confident that I will not be infected with coronavirus	174 (13.5)	412 (31.9)	548 (42.5)	156 (12.1)
I am confident that my body’s immunity can fight coronavirus infection	125 (9.7)	493 (38.2)	566 (43.9)	106 (8.2)
Controlling the spread of the COVID-19 pandemic is my responsibility	1,058 (82.0)	230 (17.8)	2 (0.2)	0 (0)
My behaviour can stop the transmission of COVID-19	970 (75.2)	313 (24.3)	6 (0.5)	1 (0.1)
Government orders to stay at home can stop the transmission of COVID-19	1,069 (82.9)	218 (16.9)	3 (0.2)	0 (0)
I will quarantine at home if I have flu symptoms like fever, cough or runny nose	926 (71.8)	286 (22.2)	61 (4.7)	17 (1.3)
Wearing protective equipment like face masks and gloves in public is simple for me	668 (51.8)	548 (42.5)	71 (5.5)	3 (0.2)
Keeping one metre physical distant from other people in public is simple for me	715 (55.4)	508 (39.4)	65 (5.0)	2 (0.2)

**Table 4:** Compliant of Malaysians on the preventive behaviours against COVID-19 as recommended by the authorities

Preventive behaviour	Frequency (%)		

	All the time	Sometimes	Not at all
I follow the order of authorities to stay at home	1,119 (86.7)	171 (13.3)	0 (0)
I obey the prohibition to stay away from crowded places	1,219 (94.5)	71 (5.5)	0 (0)
I obey the prohibition against attending public gatherings such as weddings, religious events, etc to prevent COVID-19 infection	1,287 (99.8)	3 (0.2)	0 (0)
I avoid touching my eyes, nose or mouth to prevent COVID-19 infection	809 (62.7)	468 (36.3)	13 (1.0)
I wear a face mask in public places	1,188 (92.1)	91 (7.1)	11 (0.9)
I wear a face mask when I have the flu	1,050 (81.4)	190 (14.7)	50 (3.9)
I cover my mouth with a tissue/handkerchief when sneezing or coughing	1,014 (78.6)	265 (20.5)	11 (0.9)
I wash my hands with soap after touching other people’s equipment or belongings	1,098 (85.1)	189 (14.7)	3 (0.2)
I use hand sanitiser when in contact with any objects or surfaces that have an unknown hygienic state	1,075 (83.3)	199 (15.4)	16 (1.2)
I maintain a distance of one metre from other people to prevent COVID-19 infection	1,195 (92.6)	95 (7.4)	0 (0)

**Table 5:** Distribution of total score of knowledge, attitude and preventive behaviour among Malaysians during COVID-19 pandemic

	Percentage of total score		Frequency of score above median (%)

	Mean (SD)	Median (IQR)	
Knowledge and awareness	88.9 (11.2)	88.9 (11.1)	612 (47.4)
Susceptibility and intention attitude	84.5 (7.8)	84.4 (9.4)	595 (46.1)
Preventive behaviour	92.4 (9.1)	95.0 (10.0)	516 (40.0)

**Table 6:** Association between sociodemography characteristics and knowledge on COVID-19

Variables	Frequency (%)		Crude OR (95% CI)	*P*-value*

	Good level *n* = 612	Poor level *n* = 678		
Age (years)	43.12 (10.10)#	41.28 (10.30)#	0.98 (0.97, 0.99)	0.001
Sex				
Male	159 (26.0)	175 (25.8)	1	
Female	453 (74.0)	503 (74.2)	1.01 (0.79, 1.30)	0.945
Ethnicity				
Others	68 (11.1)	93 (13.7)	1	
Malay	500 (81.7)	553 (81.6)	0.81 (0.58, 1.13)	0.215
Chinese	31 (5.1)	25 (3.7)	0.59 (0.32, 1.09)	0.091
Indian	13 (2.1)	7 (1.0)	0.39 (0.15, 1.04)	0.060
Marital status				
Single	96 (15.7)	118 (17.4)	1	
Married	486 (79.4)	527 (77.7)	0.88 (0.66, 1.19)	0.407
Divorced/widow	30 (4.9)	33 (4.9)	0.90 (0.51, 1.57)	0.699
Education level				
Primary school and less	5 (0.8)	13 (1.9)	1	
High school	66 (10.8)	80 (11.8)	0.47 (0.16, 1.38)	0.167
Diploma	110 (18.0)	117 (17.3)	0.41 (0.14, 1.19)	0.100
Bachelor	343 (56.0)	363 (53.5)	0.41 (0.14, 1.15)	0.091
Master/PhD	88 (14.4)	105 (15.5)	0.46 (0.16, 1.34)	0.154
Occupation				
Self-employed/odd job/others	48 (7.8)	51 (7.5)	1	
Government sector	376 (61.4)	447 (65.9)	1.12 (0.74, 1.70)	0.598
Private sector	92 (15.0)	80 (11.8)	0.82 (0.50, 1.34)	0.428
Housewife	34 (5.6)	35 (5.2)	0.97 (0.52, 1.79)	0.920
Retired/unemployed	41 (6.7)	25 (3.7)	0.57 (0.30, 1.08)	0.083
Student	21 (3.4)	40 (5.9)	1.79 (0.93, 3.46)	0.763
Family income (RM)				
T20	316 (51.6)	310 (45.7)	1	
M40	177 (28.9)	208 (30.7)	1.37 (1.03, 1.82)	0.030
B40	119 (19.4)	160 (23.6)	1.20 (0.93, 1.54)	0.164

Notes:

* simple logistic regression;

# mean (SD)

**Table 7:** Association between sociodemography characteristics and attitude on COVID-19

Variables	Frequency (%)		Crude OR (95% CI)	*P*-value*

	Good level *n* = 595	Poor level *n* = 695		
Age (years)	41.51 (10.00)#	42.69 (10.43#	1.01 (1.00, 1.02)	0.039
Sex				
Male	137 (23.0)	197 (28.3)	1	
Female	458 (77.0)	498 (71.7)	0.76 (0.59, 0.97)	0.030
Ethnicity				
Others	88 (14.8)	73 (10.5)	1	
Malay	479 (80.5)	574 (82.6)	1.45 (1.04, 2.02)	0.030
Chinese	20 (3.4)	36 (5.2)	2.17 (1.16, 4.07)	0.016
Indian	8 (1.3)	12 (1.7)	1.81 (0.70, 4.66)	0.220
Marital status				
Single	106 (17.8)	108 (15.5)	1	
Married	460 (77.3)	553 (79.6)	1.18 (0.88, 1.59)	0.272
Divorced/widow	29 (4.9)	34 (4.9)	1.15 (0.65, 2.02)	0.625
Education level				
Primary school and less	2 (0.3)	16 (2.3)	1	
High school	58 (9.7)	88 (12.7)	0.19 (0.04, 0.86)	0.031
Diploma	99 (16.6)	128 (18.4)	0.16 (0.04, 0.72)	0.017
Bachelor	343 (57.6)	363 (52.2)	0.13 (0.03, 0.58)	0.007
Master/PhD	93 (15.6)	100 (14.4)	0.13 (0.03, 0.60)	0.009
Occupation				
Self-employed/odd job/others	43 (7.2)	56 (8.1)	1	
Government sector	396 (66.6)	427 (61.4)	0.83 (0.54, 1.26)	0.379
Private sector	80 (13.4)	92 (13.2)	0.88 (0.54, 1.45)	0.624
Housewife	29 (4.9)	40 (5.8)	1.06 (0.57, 1.97)	0.856
Retired/unemployed	22 (3.7)	44 (6.3)	1.54 (0.80, 2.94)	0.194
Student	25 (4.2)	36 (5.2)	1.11 (0.58, 2.11)	0.761
Family income (RM)				
T20	311 (52.3)	315 (45.3)	1	
M40	172 (28.9)	213 (30.6)	1.47 (1.11, 1.96)	0.008
B40	112 (18.8)	167 (24.0)	1.22 (0.95, 1.58)	0.122

Notes:

* simple logistic regression;

# mean (SD)

**Table 8:** Association between sociodemography characteristics and preventive behaviour, and practices on COVID-19

Variables	Frequency (%)		Crude OR (95% CI)	*P-v*alue*

	Good level *n* = 516	Poor level *n* = 774		
Age (years)	42.82 (9.48)#	41.71 (10.55)#	0.99 (0.98, 1.00)	0.057
Sex				
Male	127 (24.6)	207 (26.7)	1	
Female	389 (75.4)	567 (73.3)	0.89 (0.69, 1.16)	0.392
Ethnicity				
Others	78 (15.1)	83 (10.7)	1	
Malay	415 (80.4)	638 (82.4)	1.45 (1.04, 2.02)	0.030
Chinese	13 (2.5)	43 (5.6)	3.11 (1.55, 6.22)	0.001
Indian	10 (1.9)	10 (1.3)	0.94 (0.37, 2.38)	0.940
Marital status				
Single	63 (12.2)	151 (19.5)	1	
Married	430 (83.3)	583 (75.3)	0.57 (0.41, 0.78)	< 0.001
Divorced/widow	23 (4.5)	40 (5.2)	0.73 (0.40, 1.31)	0.288
Education level				
Primary school and less	8 (1.6)	10 (1.3)	1	
High school	53 (10.3)	93 (12.0)	1.40 (0.52, 3.77)	0.501
Diploma	91 (17.6)	136 (17.6)	1.20 (0.45, 3.14)	0.717
Bachelor	301 (58.3)	405 (52.3)	1.08 (0.42, 2.76)	0.878
Master/PhD	63 (12.2)	130 (16.8)	1.65 (0.62, 4.39)	0.315
Occupation				
Self-employed/odd job/others	33 (6.4)	66 (8.5)	1	
Government sector	365 (70.7)	458 (59.2)	0.63 (0.40, 0.97)	0.038
Private sector	60 (11.6)	112 (14.5)	0.93 (0.55, 1.57)	0.796
Housewife	20 (3.9)	49 (6.3)	1.23 (0.63, 2.39)	0.551
Retired/unemployed	25 (4.8)	41 (5.3)	0.82 (0.43, 1.57)	0.549
Student	13 (2.5)	48 (6.2)	1.85 (0.88, 3.88)	0.105
Family income (RM)				
T20	260 (50.4)	366 (47.3)	1	
M40	153 (29.7)	232 (30.0)	1.21 (0.91, 1.62)	0.191
B40	103 (20.0)	176 (22.7)	1.08 (0.83, 1.40)	0.573

Notes:

* simple logistic regression;

# mean (SD)

**Table 9:** Association between sociodemography characteristics and poor of knowledge, attitude and behaviour on COVID-19

Variables	AOR (95% CI)#		

	Knowledge	Attitude	Behaviour
Model summary			
Cox & Snell *R*^2 ^*P- *value	0.014	0.013	0.025
Hosmer-Lemeshow test			
* P-*value	0.786	0.166	0.992
Age (years)	0.98 (0.97, 0.99)*	1.01 (1.00, 1.02)*	
Sex			
Female		1	
Male		1.35 (1.05, 1.74)*	
Ethnicity			
Others		1	1
Malay		1.41 (1.01, 1.98)*	1.28 (0.91, 1.80)
Chinese		2.19 (1.19, 4.13)*	2.80 (1.39, 5.61)*
Indian		1.77 (0.68, 4.58)	0.92 (0.36, 2.34)
Occupation			
Self-employed/odd job/others	1		1
Government sector	1.14 (0.75, 1.74)		0.64 (0.41, 1.00)
Private sector	0.78 (0.47, 1.28)		0.94 (0.56, 1.58)
Housewife	0.96 (0.52, 1.78)		1.24 (0.64, 2.42)
Retired/unemployed	0.72 (0.37, 1.41)		0.82 (0.43, 1.57)
Student	1.35 (0.67, 2.73)		1.77 (0.84, 3.73)

Notes:

* *P* < 0.05;

# multiple logistic regression; assumptions of linearity, multicollinearity and interaction were checked and found none
